# Nomogram to predict overall survival based on the log odds of positive lymph nodes for patients with endometrial carcinosarcoma after surgery

**DOI:** 10.1186/s12885-021-08888-0

**Published:** 2021-10-27

**Authors:** Linzhi Gao, Jun Lyu, Xiaoya Luo, Dong Zhang, Guifang Jiang, Xian Zhang, Xuesong Gao, Shaolie Zheng, Xiaoyu Wang, Yuan Shen

**Affiliations:** 1 Department of Gynaecology, The First Affiliated Hospital of Jinan University, 613 Whampoa Avenue, Tianhe District, Guangzhou, 510632 China; 2 Department of Clinical Research, The First Affiliated Hospital of Jinan University, 613 Whampoa Avenue, Tianhe District, Guangzhou, China; 3 Department of Gynecology and Obstetrics, The Affiliated Shunde Hospital of Jinan University, 50 East Guizhou Avenue, Shunde District, Foshan, China

**Keywords:** Endometrial carcinosarcoma, Overall survival rate, Log odds of positive lymph nodes, SEER, Nomogram

## Abstract

**Purpose:**

Aims to compare the prognostic performance of the number of positive lymph nodes (PLNN), lymph node ratio (LNR) and log odds of metastatic lymph nodes (LODDS) and establish a prognostic nomogram to predict overall survival (OS) rate for patients with endometrial carcinosarcoma (ECS).

**Methods:**

Patients were retrospectively obtained from Surveillance, Epidemiology and End Results (SEER) database from 2004 to 2015. The prognostic value of PLNN, LNR and LODDS were assessed. A prediction model for OS was established based on univariate and multivariate analysis of clinical and demographic characteristics of ECS patients. The clinical practical usefulness of the prediction model was valued by decision curve analysis (DCA) through quantifying its net benefits.

**Results:**

The OS prediction accuracy of LODDS for ECS is better than that of PLNN and LNR. Five factors, age, tumor size, 2009 FIGO, LODDS and peritoneal cytology, were independent prognostic factors of OS. The C-index of the nomogram was 0.743 in the training cohort. The AUCs were 0.740, 0.682 and 0.660 for predicting 1-, 3- and 5-year OS, respectively. The calibration plots and DCA showed good clinical applicability of the nomogram, which is better than 2009 FIGO staging system. These results were verified in the validation cohort. A risk classification system was built that could classify ECS patients into three risk groups. The Kaplan-Meier curves showed that OS in the different groups was accurately differentiated by the risk classification system and performed much better than FIGO 2009.

**Conclusion:**

Our results indicated that LODDS was an independent prognostic indicator for ECS patients, with better predictive efficiency than PLNN and LNR. A novel prognostic nomogram for predicting the OS rate of ECS patients was established based on the population in the SEER database. Our nomogram based on LODDS has a more accurate and convenient value for predicting the OS of ECS patients than the FIGO staging system alone.

**Supplementary Information:**

The online version contains supplementary material available at 10.1186/s12885-021-08888-0.

## Introduction

Endometrial cancer is one of the most common gynecologic malignancies in the world. More than 65,000 new cases were confirmed in the United States in 2020 [1]. Endometrial carcinosarcoma (ECS), composed of epithelial and mesenchymal cells, is a rare and aggressive solid malignant tumor, which accounts for less than 5% of uterine malignancies, but about 15% of uterine cancer deaths are related to ECS [2, 3]. Recent studies have shown that ECS is more prone to lymph node (LN) metastasis and recurrence after surgery. The foundation of therapy for ECS is surgical resection, including total abdominal hysterectomy, bilateral salpingo-oophorectomy, and lymph node dissection, with or without combination chemoradiation [4, 5]. Although adopting this aggressive surgical method, the local area recurrence rate is as high as 60%. The International Federation of Gynecology and Obstetrics (FIGO) recommended that ECS use the same staging system as endometrial adenocarcinoma, namely the 2009 FIGO staging system, and pointed out that the clinical pathological disease staging at the time of diagnosis is an important factor affecting the prognosis [6]. However, up to 30% of ECS patients may have extra-uterine metastases at the time of onset, resulting in a significantly worse prognosis than endometrial adenocarcinoma [7]. Studies pointed out that the 5-year overall survival rate for stage I or II ECS patients is 30–45%, and the 5-year overall survival rate for stage III or IV ECS patients is 0–10% [2, 6, 7].

Some studies have demonstrated that the postoperative survival rate is not only affected by the overall LN status (i.e., no metastases versus metastases), but also by the number of metastatic LNs [8, 9]. Therefore, adequate LN histopathological evaluation is essential to predict the prognosis of ECS. However, the current 2009 FIGO staging system of endometrial cancer is only based on the anatomical location of metastatic LN metastasis, without considering the number of metastatic LNs, which may limit its prognostic accuracy [10]. Many studies have referred that the LN status of several solid tumors usually depends on the anatomical location and the number of metastatic LNs [11]. In recent years, many studies have shown that there is a significant correlation between different LN staging systems and patient survival outcomes, including the number of positive lymph nodes (PLNN), lymph node ratio (LNR), and log odds of positive lymph nodes (LODDS) [12–14].

LNR is the ratio of the number of positive LNs to the total number of resected LNs [13]. It was reported that LNR provides important guidance regarding the survival of patients with gastric adenocarcinoma, which have shown its superiority in guiding the prognosis over PLNN [12]. LODDS, defined as the logarithm of the ratio of the number of positive and negative LNs, has been applied to predict the prognosis of several tumors. When the number of LN removed is insufficient, the algorithm can stratify patients according to different prognosis [15]. At present, there are few studies on the value of different LN staging systems in predicting the prognosis of ECS, and the most appropriate way for predicting the prognosis of ECS remains unclear.

The overall prognosis of women with ECS is dismal. The survival outcomes of women with ECS are even worse than other types of high-grade endometrial cancers [16, 17]. Therefore, the purpose of this study was to compare the prognostic performance f PLNN, LNR and LODDS and establish a prognostic nomogram to predict overall survival (OS) rate for patients with ECS based on the population derived from Surveillance, Epidemiology and End Results (SEER) database.

## Methods

### Patient inclusion

Patients diagnosed with ECS between 2004 and 2015 were retrieved from the Surveillance, Epidemiology, and End Results (SEER) database (SEER*Stat version 8.3.8). For data collection, we limited Primary Site: the International Classification of Diseases for Oncology, third edition (ICD-O-3) C54.1. And select only malignant cancers and known age. In total, 99,177 records were collected.

**Fig. 1:**
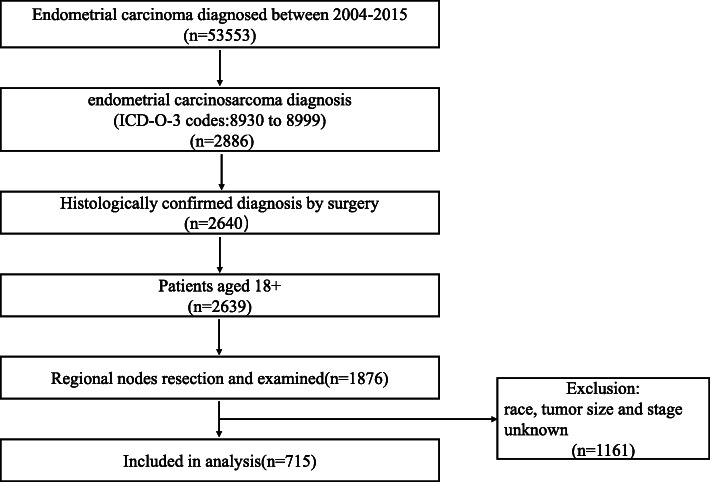
Flow chart for creation of the patient data

The inclusion criteria including: (1) patients diagnosed with ECS between 2004 and 2015; (2) patients with a histologic diagnosis of ECS (ICD-O-3:8930 to 8999); (3) patients who were 18 years old or older at diagnosis; (4) patients with regional nodes resection and examined after surgery. The exclusion criteria ruled out patients with inadequate information on race, tumor size, tumor extension, the seventh edition of the AJCC stage, patients with inadequate information on LNs (including examined LNs and positive LNs); and absent information on survival months or cause of death. Finally, based on the aforementioned criteria, a total of 715 patients were included and the data process flowchart was presented in Fig. 1. Afterwards, the patients assigned to the training cohort and the validation cohort with a portion of 7:3, using a random sampling method.

### Characteristics

The data of clinical characteristics including year of diagnosis, age, race, metastatic status, histologic grade, tumor size, cause of death, peritoneal cytology status, the seventh edition of the AJCC staging system, the total amount of lymph nodes retrieved, the amount of metastatic lymph nodes, survival time, and survival status were collected from the SEER database. The original staging information of ECS in the SEER database is the seventh edition of the AJCC staging system. On the basis of the 2009 FIGO staging system, we transformed the seventh edition of the AJCC staging system to 2009 FIGO in this study. PLNN represents the numbers of positive lymph nodes. LNR is the ratio of the number of positive LNs to the total number of resected LNs. LODDS, defined as the logarithm of the ratio of the number of positive and negative LNs.

The main endpoint was overall survival (OS) rate which was calculated from the date of diagnosis to the date of death from any cause. Optimal cutoff values were determined using X-tile software. Based on the optimal cut-off value, PLNN, LNR, and LODDS was calculated into categorized variables. Tumor size was divided into ≤58 mm, and > 58 mm groups. PLNN was classified into two group: namely PLNN1 (=0) and PLNN2 (> 0). LNR was divided into two categories, namely LNR1 (≤0.03448276) and LNR2 (> 0.03448276). The LODDS was divided into two subgroups, namely LODDS1 (LODDS≤ − 0.9199705), LODDS2 (LODDS> − 0.9199705).

We obtained approval to access the SEER of the National Cancer Institute in the United States using the reference number 20256-Nov2019.

### Statistical analysis

#### Development of the model

Relations to OS were evaluated with a univariable analysis according to the Kaplan–Meier approach and using the log-rank test to assess statistically significant differences among groups. To predict 1-, 3- and 5-year OS, a multivariate cox proportional hazards model was performed, which included the relevant predictors in univariate analysis (*P* < 0.1) (Table 2). The multivariate analysis was applied to generate the nomogram based on the R software. We assessed the predictive performance of the nomogram by evaluating the concordance index (C-index), the area under the receiver operating characteristic (ROC) curve (AUC), the Akaike information criterion (AIC) and calibration plots (comparing the survival probability predicted by the nomogram with the observed value by Kaplan–Meier analysis). A smaller AIC value indicated a better model for predicting outcome. Backward stepwise selection was performed to determine independent covariates [12–15]. Variables entered into the model were age, tumor size, 2009 FIGO, LODDS and peritoneal cytology. Variables were eliminated from the model if their removal actually improved the overall quality of the model (as measured by AIC). Additionally, according to the total score of each patient in the training cohort by using the nomogram, all patients were divided into three prognostic groups (namely low-, intermediate-, and high-risk groups) with similar number of patients to establish a risk classification system. Kaplan-Meier curve and log-rank test were used to illustrate and compare the OS of patients in different risk groups.

#### Validation of the model

The nomogram was confirmed using the validation cohort of 216 patients. A bootstrap re-sampling method to obtain relatively unbiased estimates (1000 repetitions) was used for external validation. For each group of 1000 bootstrap samples, the model was refitted and tested against the observed sample to estimate the predictive accuracy and bias [6, 12, 13].

Additionally, decision curve analysis (DCA) assisted in confirming the threshold probability range of the nomogram, which was compared with the 2009 FIGO staging system. Besides, the predictive efficiency of PLNN, LNR, and LODDS were compared using the C-index, AIC, and AUC [12–15].

Descriptive statistics are described as mean ± standard deviation(SD)for continuous variables and number for categorical variables. A chi-square test was used for the analysis of all categorical data. The Kruskal–Wallis H test or Wilcoxon test was used for the analysis of continuous variables. Bonferroni-adjusted significance tests were applied for pairwise comparisons. The Kaplan–Meier method and the log-rank test were used to construct and compare the survival curves, respectively. Statistical analysis was carried out with SPSS (Statistical Package for the Social Sciences) for Windows, version 22, and R 3.6.3 software (http://www.r-project.org). A *p* < 0.1 was chosen as the criterion for removing a variable from the multivariate Cox proportional hazards model, and a *p* < 0.05 was considered significant for all other tests.

## Results

### Patient characteristics and survival outcomes

The study enrolled 715 patients with ECS diagnosed from 2004 to 2015 in the SEER database. These patients randomly divided into a training cohort and a validation cohort by a ratio of 7:3. The clinical and demographic characteristics of the involved patients are summarized in Table 1. The mean age of these patients was 63.38 years (range 21 to 85 years) in the whole population, 63.52 years (range 24 to 85 years) in the training cohort, and 63.05 years (range 21 to 85 years) in the validation cohort. Among the whole population, there were 385 (53.85%) patients diagnosed with I stage, 55 (7.69%) patients diagnosed with II stage, 196 (27.41%) patients diagnosed with III stage, 79 (11.05%) patients diagnosed with IV stage. Moreover, the mean PLNN were 1.08 ± 2.23, the mean LNR were 0.81 ± 0.73, and the mean LODDS were 0.048 ± 1.71. In order to compare different LN staging systems comprehensively and reasonably, we grouped continuous variables of the PLNN, LNR and LODDS schemes into two classification levels according to best cut-off points. PLNN was classified into two group: 205 (29%) in PLNN1 (=0) and 510 (71%) in PLNN2 (> 0). LNR classification was determined: 207 (29%) in LNR1 (≤ 0.03448276), 508 (71%) in LNR2 (> 0.03448276). For the LODDS system, 222 (31%) patients were in the LODDS1 group (LODDS≤ − 0.9199705), and 493 (69%) patients were in the LODDS2 group (LODDS> − 0.9199705). At the last follow-up, 303 patients (42%) died. Only 33 patients (11% of all deaths) died of causes other than ECS. The median OS in the whole population (*n* = 715) was 51 months.

**Table 1:** Clinical and demographic characteristics of patients with ESC from SEER database, 2004–2015

Variables	All subjects (*n* = 715)	Training cohort (*n* = 499)	Validation cohort (*n* = 216)	*P* value
Age (years)	63.38 ± 11.43	63.52 ± 11.31	63.05 ± 11.70	0.469
Race				0.675
White	483	341	142	
Black	144	100	44	
Other	88	58	30	
Tumor size (mm)				0.354
≤ 58 mm	342	233	109	
>58 mm	373	266	107	
FIGO2009				0.550
I	385	266	119	
II	55	36	19	
III	196	144	52	
IV	79	53	26	
Examined nodes	17.63 ± 12.97	18.75 ± 13.33	18.36 ± 12.61	0.486
PLNN				0.709
1	205	141	64	
2	510	358	152	
LNR				0.792
1	207	143	64	
2	508	356	152	
LODDS				0.991
1	222	155	67	
2	493	344	149	
Histology grade				0.751
I	19	12	7	
II	53	39	14	
III	256	184	72	
IV	179	124	55	
unknow	208			
Peritoneal Cytology				0.837
positive	101	68	33	
negative	444	311	133	
unknow	170	120	50	

### Prognostic factors of OS

Table 2 summarizes univariate and multivariate analyses of the training cohort. By using univariate cox regression analysis, age, tumor size, 2009 FIGO, examined nodes, PLNN, LNR, LODDS and peritoneal cytology were associated with OS (*P* < 0.1). According to multivariate cox regression analysis, five parameters, age, tumor size, 2009 FIGO, LODDS and peritoneal cytology were defined as independent prognostic factors of OS (P < 0.1). Advancing age, tumor size> 58 mm, higher 2009 FIGO stage, LODDS> − 0.9199705, and positive peritoneal cytology were associated with a decreased OS rate.

**Table 2:** Univariate and multivariate analysis of OS in training cohort (*N* = 499)

	Univariate analysis			Multivariate analysis		
Variables	HR	95% CI	*P* value	HR	95% CI	*P* value
Age (years)	1.03	1.017–1.043	< 0.001	1.024	1.01–1.037	< 0.001
Race						
White			Reference			Reference
Black	1.621	1.177–2.232	0.003	1.353	0.961–1.904	0.083
Other	1.279	0.84–1.948	0.251	1.348	0.859–2.116	0.194
Tumor size (mm)						
≤ 58 mm			Reference			Reference
>58 mm	2.209	1.653–2.953	< 0.001	1.580	1.161–2.151	0.004
FIGO2009 (1,2,3,4)						
I			Reference			Reference
II	2.043	1.203–3.469	0.008	2.013	1.173–3.453	0.011
III	2.629	1.91–3.618	< 0.001	2.199	1.565–3.089	< 0.001
IV	3.642	0.889–14.918	0.072	2.281	0.545–9.537	0.259
Examined nodes	0.982	0.971–0.994	0.003	0.986	0.974–0.998	0.019
PLNN						
1			Reference			Reference
2	2.181	1.485–3.205	< 0.001	3.490	0.449–27.134	0.232
LNR						
1			Reference			Reference
2	2.132	1.458–3.116	< 0.001	0.152	0.015–1.517	0.108
LODDS						
1			Reference			Reference
2	2.178	1.509–3.142	< 0.001	3.164	0.991–10.106	0.052
Histology grade						
I			Reference			Reference
II	0.604	0.111–3.301	0.561	0.589	0.107–3.244	0.543
III	3.743	0.922–15.205	0.065	2.250	0.547–9.252	0.261
IV	3.642	0.889–14.918	0.072	2.281	0.545–9.537	0.259
unknow	2.843	0.694–11.653	0.147	1.883	0.453–7.822	0.384
Peritoneal Cytology						
negative			Reference			Reference
positive	2.617	1.85–3.701	< 0.001	1.395	0.959–2.03	0.081
unknow	2.047	1.493–2.807	< 0.001	1.593	1.14–2.227	0.006

Table 2 summarizes the comparison of the predictive value of three LN staging systems. AIC of predicted OS rate for PLNN, LNR and LODDS were 2439.226, 2439.998 and 2437.721. C-index of PLNN, LNR and LODDS were 0.563, 0.562, 0.570, respectively (Table 3).

**Table 3:** Evaluation of the prognostic value of the three different LN staging systems

			AUC		
Variables(OS)	C-index	AIC	1-year survival	3-year survival	5-year survival
PLNN (categorical)	0.563	2439.226	0.569	0.582	0.582
LNR (categorical)	0.562	2439.998	0.564	0.581	0.581
LODDS (categorical)	0.57	2437.721	0.578	0.586	0.586

### Construction and validation of the Nomogram for OS

**Fig. 2:**
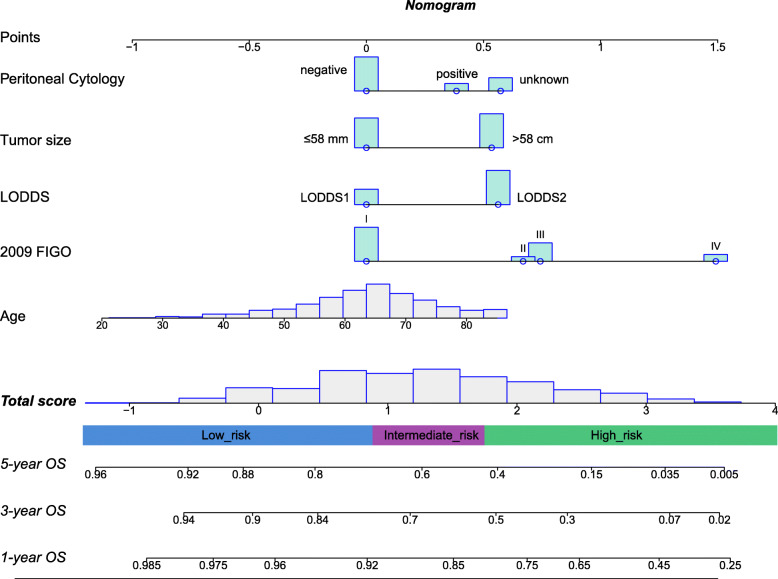
Nomogram for prognostic prediction of a patient with ECS. The probability of 1-, 3- and 5-year OS rate is calculated by drawing a line to the point on the axis for each of the following variables: age, tumor size, 2009 FIGO, LODDS and peritoneal cytology. The points for each variable are summed and located on the total points line. Next, a vertical line is projected from the total points line to the predicted probability bottom scale to obtain the individual 1-, 3- and 5-year OS axes

On the basis of the univariate and multivariate cox regression analyses we showed above, a nomogram incorporating the significant risk factors was established to predict 1-, 3- and 5-year OS rates of ECS patients (Fig. 2). And the Additiona file 1: Fig.S1 provides a direct vision for daily use of the model. For predicting OS rate, the c-statistic of this nomogram in the training cohort was 0.743 [95% confidence interval (95% CI), 0.718–0.769] compared with 0.674 (95% CI, 0.647–0.701) for 2009 FIGO staging system. The c-statistic of the nomogram in the validation cohort was 0.735 (95% CI, 0.717–0.753), compared with 0.669 (95% CI, 0.651–0.687) for the FIGO 2009 staging system.

**Fig. 3:**
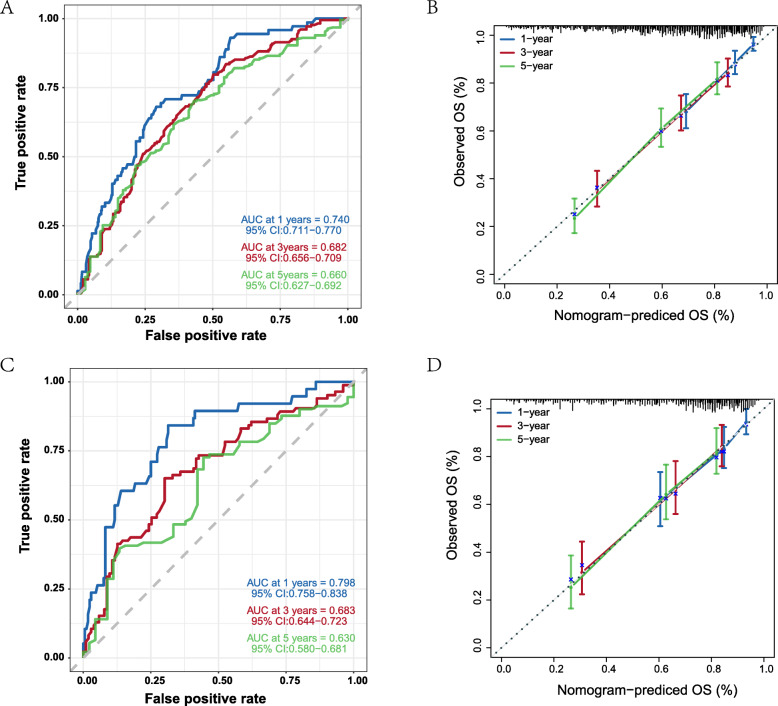
ROCs curve and Calibration plots for nomogram in training cohort(A/B) and in validation cohort(C/D). A Discrimination for the training cohort. B Calibration for the training cohort. C Discrimination for the validation cohort. B Calibration for the validation cohort

Furthermore, the predictive performance of our nomogram was calculated by time ROC curve (Fig. 3A, C). The AUC values in the training cohort were 0.740, 0.682 and 0.660 for 1-, 3- and 5-year OS rates and in the validation cohort were 0.798, 0.683 and 0.630, both indicating good statistic power of the nomogram.

Besides, calibration was good in both training and validation cohort (Fig. 3B, D). These results indicated that compared with the FIGO 2009 staging system, our nomogram demonstrated better discrimination and prognostic prediction capabilities.

### Clinical value of nomogram

**Fig. 4:**
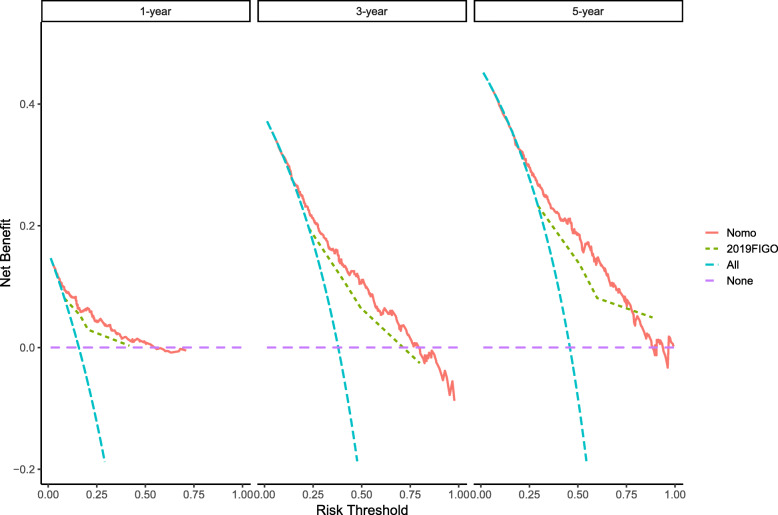
DCA between nomogram and the FIGO 2009 staging system for 1-, 3-, and 5-year OS. X-axis represents risk threshold, and Y-axis measures net benefit. The horizontal line along X-axis assumes that overall death occurred in no patients, whereas blue dashed line assumes that all patients will have overall death at a specific threshold probability. Red dashed line represents nomogram. Green dashed line represents 2009 FIGO staging system

According to DCA, compared with the FIGO 2009 staging system, the nomogram demonstrated more net benefit across the range of decision threshold probabilities (Fig. 4). Most importantly, patients can benefit more from the nomogram to predict individual survival outcomes.

### Risk classification system of Nomogram

**Fig. 5:**
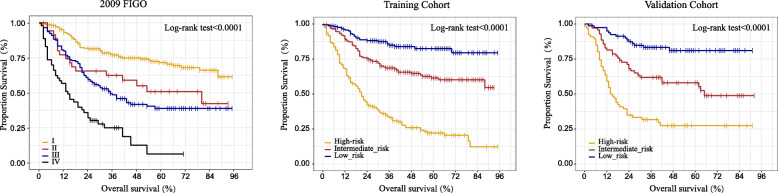
A Kaplan-Meier Curves of Overall survival for 2009 FIGO staging system in the whole cohort. B Kaplan-Meier Curves of Overall survival for patients in the low-, intermediate-, and high-risk groups in the training cohort. C Kaplan-Meier Curves of Overall survival for patients in the low-, intermediate-, and high-risk groups in the validation cohort

In addition to the nomogram, a risk classification system for OS was also developed according to the total scores of each patient in the training cohort produced by the nomogram to divide all patients into three prognostic groups, with a similar number of cases per group. Based on the novel classification system, all patients were classified into the low-risk (166/499, 33.3%; score − 1.025 to 0.837), intermediate-risk (167/499, 33.4%; score 0.837 to1.655), or high-risk groups (166/499, 33.1%; score 1.655 to 4) (Fig. 2). The Kaplan–Meier curves showed that OS in the different groups was accurately differentiated by the risk classification system (Fig. 5). and performed much better than FIGO 2009.

## Discussion

ECS is a rare tumor with a poor prognosis due to its extremely aggressive behavior [3]. An accurate staging system of predict survival is pivotal to help guide treatment selection and judgment of the prognosis of patients with ECS. According to the 2009 FIGO staging system, the most widely utilized staging system in endometrial tumors, it considers only the anatomic locations of positive LNs and lacks consideration of the number of positive LNs, resulting in decreased prognostic value [10]. Therefore, in our study, a prognostic model was established and validated to predict OS of ECS to help guide the management of postoperative ECS patients. The nomogram was developed in a training cohort including 499 patients and tested on an external independent validation cohort including 216 patients derived from SEER. Five variables, age, tumor size, 2009 FIGO, LODDS and peritoneal cytology, were selected by stepwise regression based on the minimum value of AIC and merged into the nomogram. The verification of the nomogram indicated that it has good discrimination and calibration capabilities. Previous studies have raised some factors that may affect the OS of ECS patients, such as advancing age, stage and lymph node metastasis [2, 6, 8, 18–21]. These factors have been fully considered in this study. The nomogram clearly outperformed the FIGO 2009 staging system and could inform patients about their prognosis and perform better predictive value.

Recently, because of the close relation between LN status and prognosis in many tumors, many studies were conducted to explore a brilliant LN staging system [13, 22, 23]. Previous studies have provided different results for the evaluation of these different staging systems in different tumors, some of which support the prognostic ability of the LNR staging system, while others advocate the use of the LODDS staging system [13, 24–27].. In this study, we compared the predictive abilities of PLNN, LNR and LODDS. The results showed that by comparing the C index, AUC and AIC of the three lymph node staging systems, LODDS has a slightly better prognostic indicator for predicting OS of ECS patients. In addition, through multivariate cox analysis, our study indicated that LODDS was an independent prognostic determinant that affects the prognosis of ECS patients. On the other hand, not only the absolute number of positive lymph nodes, but also the number of negative lymph nodes were considered on LODDS. Therefore, LODDS has better ability of discrimination, especially in patients with no lymph node involvement or all lymph node involvement. To the best of our knowledge, this was the first study to evaluate the prognostic ability of different LN staging systems for ECS patients.

According to our results, patients in the group of advancing age, tumor size> 58 mm, and positive peritoneal cytology were at a significantly worse prognosis than others in OS rate. It was previously reported that compared with the small-size group, the large-size group was more tend to lymph node metastasis, distant metastasis and aggressive growth characterized, all of which were related with a poor prognosis [28, 29]. According to the current staging system (2009 FIGO), malignant peritoneal cytology is not included as a basis of staging factors, while malignant peritoneal cytology has been reported to be strongly related with an increased risk of all-cause mortality of ECS patients [30].

DCA is a practical tool in determining the effectiveness of model-based clinical decisions, which can directly provide with useful clinical information [31]. We applied DCA to estimate the effectiveness of threshold probability–based prediction models in clinical practice., which demonstrated that compared with the 2009 FIGO alone, the nomogram has more advantages. Therefore, based on the SEER database, we established a nomogram based on the LODDS system to predict the OS rate of ECS patients. Compared with FIGO 2009 staging, our nomogram shows higher accuracy and relatively better prognostic judgment. Furthermore, a novel risk classification was established on basis of the predictive scores calculated by the nomogram, which classified all patients into three different prognostic groups.

It should be pointed out that our research has some limitations. First, familial endometrial carcinoma such as Lynch syndrome, an autosomal dominant susceptibility disease, which is not enrolled in the SEER database. Second, the absence of detailed individual information on chemotherapy and radiotherapy or any other treatment before surgery, which may be relevant factors that are not included in the model. Third, though SEER is a huge population-based database, retrospective data had an inherent bias and lack of external data from different cancer centers to validate the nomogram model. Furthermore, because of the characteristics of the multicentric, retrospective entry of pathology data without central pathology review in the SEER database, there could be certain deviation of diagnosis [32]. Future well-designed studies could improve the nomogram by incorporating these factors based on their predictive power.

## Conclusions

Our results indicated that LODDS was an independent prognostic indicator for ECS patients, with better predictive efficiency than PLNN and LNR. A novel prognostic nomogram for predicting the OS rate of ECS patients was established based on the population in the SEER database. Our nomogram based on LODDS has a more accurate and convenient value for predicting the OS of ECS patients than the FIGO staging system alone.

## Supplementary Information

**Additional file 1 Figure S1** Nomogram for prognostic prediction of a patient with ECS. The patient #2 is illustrated in the nomogram by mapping its values to the covariate scales. The probabilities of 1-, 3-, 5-year OS are estimated to be 0.958, 0.878, 0.843.

## Data Availability

We obtained approval to access the SEER of the National Cancer Institute in the United States using the reference number 20256-Nov2019. Data in this manuscript is available in SEER database(https://seer.cancer.gov/).

## Abbreviations

OS: Overall survival rate
LN: Lymph node
PLNN: Positive lymph nodes
LNR: Lymph node ratio
LODDS: log odds of metastatic lymph nodes
ECS: Endometrial carcinosarcoma
SEER: Surveillance, Epidemiology and End Results database
FIGO: The International Federation of Gynecology and Obstetrics
EA: endometrial adenocarcinomas.
C-index: concordance index
ROC: the receiver operating characteristic curve
AUC: the area under the receiver operating characteristic curve
AIC: the Akaike information criterion
DCA: Decision curve analysis
95% CI: 95% confidence interval
