# Supplementary figures and images for: Nomogram to predict overall survival based on the log odds of positive lymph nodes for patients with endometrial carcinosarcoma after surgery
Source: BMC Cancer. 2021 Oct 27;21:1149. doi: 10.1186/s12885-021-08888-0 (PMC8549209; doi:10.1186/s12885-021-08888-0)

# Nomogram

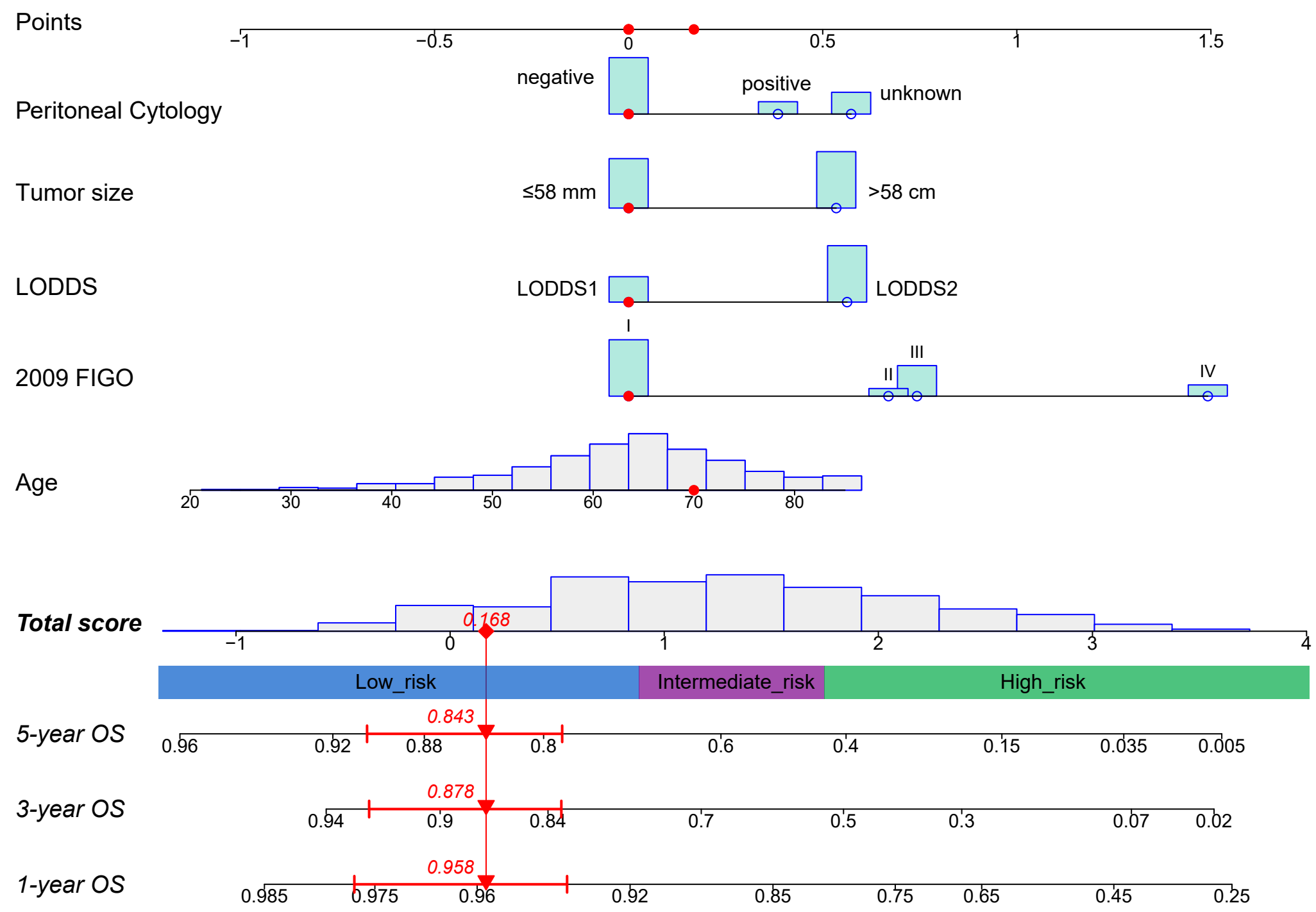

Supplement: Supplementary file 1 — Additional file 1 Figure S1 Nomogram for prognostic prediction of a patient with ECS. The patient #2 is illustrated in the nomogram by mapping its values to the covariate scales. The probabilities of 1-, 3-, 5-year OS are estimated to be 0.958, 0.878, 0.843. [file 12885_2021_8888_MOESM1_ESM.pdf]
